# Work Engagement and Flourishing at Work Among Nuns: The Moderating Role of Human Values

**DOI:** 10.3389/fpsyg.2018.01874

**Published:** 2018-09-28

**Authors:** Antonio Ariza-Montes, Horacio Molina-Sánchez, Jesús Ramirez-Sobrino, Gabriele Giorgi

**Affiliations:** ^1^Department of Management, Universidad Loyola Andalucía, Seville, Spain; ^2^Department of Business Administration, Universidad Autónoma de Chile, Santiago, Chile; ^3^Department of Psychology, Università Europea di Roma, Rome, Italy

**Keywords:** work engagement, flourishing, human values, nuns, faith-based organizations

## Abstract

Faith-based organizations are a key player in major sectors of activity for maintaining the welfare state, including health, education, and social services. This paper uses a multivariate regression model in an attempt to identify the factors that affect the relationship between work engagement and flourishing. The paper also discusses the empirical research gap that has been identified in the literature about the moderated effect of human values on this relationship. This study is based on a sample of 142 nuns of a congregation belonging to a religious organization with an international scope and a Catholic inspiration. The case of religious women who have chosen to live a life consistent with the charism of the congregation constitutes a paradigmatic and unique environment to investigate the potential alignment of personal values with professional activity. This work unveils two main findings. First, the more engaged nuns are in their work (social action to serve the poorest and most disadvantaged people), the more they flourish in their working environment and in their personal lives. Second, Schwartz’s values reinforce the relationship between the professional role (work engagement) and the personal role (flourishing at work). In conclusion, flourishing at work could be improved through work engagement and this relationship is moderated by human values. These results add insights to better know the relationship among life and work domains.

## Introduction

Researchers on vocational interests are convinced that human beings flourish in work environments that are characterized by a good fit between job characteristics and personal competences (Holland, 1996). Ehrhart and Makransky (2007) point out that person-job fit positively influences individuals and their environment, for example, by improving their satisfaction, performance and career stability. As Hartung (2009) notes, more and more employees question the meaning of work in their lives, and in relation to the purpose of this paper, they also ask how their role as employees fits with the other roles in their life to help them experience meaning and purpose.

Researchers in different areas of knowledge, such as organizational psychology, sociology or management, have analyzed the interaction between satisfaction with work and satisfaction with life and how it conditions well-being. The relationship between the domains of work and personal life is a controversial issue. Although some authors argue that there is no interaction between the two domains (e.g., Judge and Watanabe, 1994; Georgellis and Lange, 2012), others suggest the existence of a spillover or compensation effect between them (e.g., Zedeck, 1992; Newstrom, 2007).

This research analyses the relationship between work engagement (a positive mental state of connection to work) and flourishing at work. Flourishing measures self-perceived success in relationship, self-esteem, purpose and optimism (Diener et al., 2010). The concept of flourishing at work that is used in this article is an adaptation of this scale to the labor context. This study is based on a sample of very peculiar workers: 142 consecrated members of a female congregation belonging to a religious organization with an international scope and a Catholic inspiration located in the south of Spain. As Ménard and Brunet (2011) note, religious workers constitute a particularly interesting group for studying it. As they devote their life to service, a critical issue is to understand how well-being is promoted in the workplace for this group. The case of religious women who have chosen to live a life consistent with the charism of the congregation constitutes a paradigmatic and unique environment to investigate the potential alignment of personal values with professional activity. The knowledge of the explanatory variables in such an environment may be useful to enhance this alignment in other contexts.

Very few studies have analyzed work engagement among religious leaders (Miner et al., 2015). Today, faith-based organizations are a key player in major sectors of activity for maintaining the welfare state, including health, education, and social services. In Spain, for instance, social sector entities serve 12 million people at risk of exclusion and poverty, representing 1% of GDP (Fundación PwC et al., 2013). Moreover, in 2015, Catholic social action in Spain look after 4.7 millions of people distributed in 8,966 social centers. To advance the knowledge on these organizations, this paper uses a multivariate regression model in an attempt to identify the factors that affect the relationship between work engagement and flourishing at work. After this analysis, we examine whether certain human values (security, stimulation, power, universalism, etc.) moderate the work engagement and flourishing at work relationship. For the latter purpose, this research is centered on Schwartz’s (1992) Theory of Human Values.

This article aims to provide insights into an incipient research field, the relationship between dedication to work and well-being. The paper also discusses the empirical research gap that has been identified in the literature about the moderated effect of human values on this relationship. In fact, to our knowledge, no research analyses this effect in the interaction between work engagement and flourishing, let alone in relation to the peculiarities of the consecrated members of a religious order.

The structure of the paper is as follows. For justifying our research hypotheses, Section “Literature Review and Hypothesis Development” reviews the most relevant literature on work engagement and well-being topic and particularly on religious workers. Then, we describe the methodology in Section “Methodology.” Section “Results” presents the most significant results obtained in relation to the hypotheses presented. Subsequently, the results are discussed, following the structure of our hypotheses, and the main conclusions are summarized. The last section describes the main implications and limitations of the research.

## Literature Review and Hypothesis Development

Traditionally, there has been a notorious research interest in the negative aspects of the work-life relationship (McNall et al., 2010). Authors such as Karatepe and Bekteshi (2008) and Kalliath et al. (2012) indicate that the conflict between the two domains is insurmountable, since the implication in one of the roles limits and conditions the available time that can be devoted to the other.

Based on the Conservation of Resources (COR) Theory, Halbesleben (2011) considers that the investment of energy in one of the areas is a strategic decision not only to invest in that domain but also not to invest in another domain. One of the labor factors that may exert a negative impact on different areas of private life is undoubtedly work engagement.

In contrast, another group of researchers has been trying to demonstrate the existence of positive relationships between work-life in different organizational contexts. For example, Rodríguez-Muñoz et al. (2014) discover that the effects of work engagement are not limited to the workplace but, by capillarization, are transferred to other areas of life, entering the private sphere of the employee. These authors, as Greenhaus and Powell (2006), argue that work engagement improves the quality of life outside the workplace. Therefore, work engagement generates a dual positive impact in the form of an improvement in health and good social functioning (Schaufeli et al., 2008) or a more positive attitude at home, which results in more enriching family relationships (Culbertson et al., 2012).

### Work Engagement and Well-Being

The relationship between work engagement and well-being is a special setting of the work-life balance. Bowling et al. (2010) develop a meta-analysis on this relationship. In particular, several studies link work engagement with different indicators of occupational well-being, such as involvement, job satisfaction or, among others, burnout reduction (Bakker et al., 2008).

Work engagement is a positive and persistent emotional affective state in employees that is characterized by vigor, dedication and absorption (Schaufeli et al., 2002). Vigor refers to a great willingness to devote effort to work, persisting despite of difficulties. Dedication means being heavily involved in work and experiencing a sense of enthusiasm, inspiration, pride, challenge and meaning. Finally, absorption implies being totally concentrated on and happily immersed in work in such a way that time passes quickly and ceasing work leads to displeasure.

In another vein, Diener et al. (2010) identify two dimensions on well-being. The first one is the balance between positive and negative feelings; and the second one is flourishing. Flourishing is a state of mental health characterized by positive affectivity and by the presence of high levels of subjective, psychological and social well-being (Guzmán and Gom, 2013). Flourishing is more related with eudaimonic view of well-being. The election of flourishing seems more suited to religious workers who devote their lives to spiritual aims.

Among the few studies on the relationship between work engagement and well-being, studies tend to adopt a hedonic perspective of well-being and neglect the eudaimonic aspects of well-being. As noted by Ménard and Brunet (2011), analyses should contain this dual approach. Therefore, this paper focuses on flourishing as an integrative dimension of well-being. This eudaimonic vision of well-being relegates happiness to a broader perspective, focusing on the subjective perception of different areas of personal activity, e.g., self-realization, meaning or life purpose, feelings of competence and self-esteem, and personal growth (Diener et al., 2010).

### Work-Life Relationship on Religious Workers

Robert et al. (2006) have researched about the influence of spiritual well-being on job satisfaction. They identify two dimensions on spiritual well-being: existential well-being and religious one. On the one hand, existential well-being is related to the sense of vocation and purpose, and is a concept related to flourishing for consecrated members on a religious order. On the other hand, religious well-being refers to the individual belief in God. These authors found that existential well-being strongly influenced on job satisfaction, meanwhile religious well-being had a scarce influence. However, what is the influence of work engagement on existential well-being?

Non-profit organizations seem a proper setting for studying this particular link because personal vocation is fulfilled through daily work. Dempsey and Sanders (2010) discovered on the biography of social entrepreneurs the feeling of calling to give meaning to the work. Employees in non-profits organizations normally exhibit higher satisfaction than their counterparts in for-profit entities, being attributable to non-pecuniary compensation (Benz, 2005; Ariza-Montes and Lucia-Casademunt, 2016). If the relationship between work engagement and well-being is not clear, we focus on a special vocational group where the relationship between work engagement and well-being could have special characteristics. The study of vocational workers contributes with valuable insights to understand how this link works.

In this vein, Visser et al. (2016) studied the work-life balance in expatriate workers from the humanitarian sector. In general, these employees are highly committed with the aims of their jobs and they take large seasons completely devoted to the work. The results obtained for these authors show a positive relationship among autonomy at the workplace and work-life satisfaction when trust in the managers of these organizations is high.

Among vocational workers, the research in religious ones is practically non-existent, which justifies the relevance of this study. From a scientific point of view, work engagement in nuns presents particular features. The first is spiritual, since consecrated congregation members have professed perpetual vows of unconditional commitment with a cause. The second is a more mundane explanation; in many cases, nuns live in their own workplaces (24 h a day and 7 days a week) with the direct beneficiaries of their work, mainly the elderly, the indigent and helpless children. This close coexistence with users creates feelings of familiarity. Therefore, they use fraternal terms such as “sisters” with the rest of the members of the Institution.

The influence of the religious factor is currently a hot topic for researchers of organizational behavior (Monteiro, 2015). The research even scarcer with regard to the main theme of this article, i.e., the interdependence between work and flourishing in the religious context (Johnson, 2010; Chan-Serafin et al., 2013). The singularities of this collective may show a greater overlap between personal and professional life than in other groups of people. Ellison (1991) emphasizes that individuals with an intense religious faith have higher levels of satisfaction with life, greater personal happiness, and fewer negative psychosocial consequences on a daily basis. According to this author, the religious factor can reinforce individual well-being by facilitating social integration, a personal relationship with a divine other, and existential coherence by providing the individual with patterns of religious organization and personal lifestyles. Bickerton et al. (2014) conduct a longitudinal study with a sample of Christian religious workers from Australia, and they conclude that the link with God and the divine call generates more work engagement than in other groups of people. According to these authors, spiritual resources promote work engagement by increasing the meaning of the tasks and the perceived ability to successfully perform them. In this context, individual well-being must necessarily increase. It seems obvious that engaged employees are more likely to fulfill their work than unengaged ones (Halbesleben, 2010). Park (2012) suggests that this argument is even stronger among religious workers because spiritual beliefs reinforce them.

Consequently, the first research hypothesis is as follows.

**Hypothesis 1:** Nuns’ work engagement is positively related with their flourishing at work.

The studies that analyze the work engagement and flourishing link are very scarce, and to our knowledge, we have not identified any that introduces the moderating effect of values. Human values are underlying conceptions of what is good and desirable (e.g., justice, humility, tradition, and success). Schwartz’s (2006) theory suggests the existence of 10 different and universal motivational values that act together according to a hierarchy of priorities. This system of values characterizes each individual and distinguishes among individuals. These 10 basic values are grouped into four higher-order constructs: (a) self-enhancement (integrated by values of power and achievement), (b) self-transcendence (benevolence and universalism), (c) openness to change (hedonism, stimulation and self-direction), and (d) conservation (tradition, conformity, and security). These four typologies are grouped into two large bipolar dimensions: individualism-collectivism versus change-continuity. Thus, while self-enhancement overrides the particular interests of individuals and the development of their maximum potential, self-transcendence refers to a greater concern for the well-being of others. Likewise, while openness to change prompts action, living new experiences and thinking independently, conservation predisposes individuals to protect and maintain what they already possess, e.g., self-regulation, order, and resistance to anything that implies change.

Schwartz (1994) notes that values associate with what is important for life and defines them as desirable, transcendent goals that vary in importance and cement the principles that guide the life of a person or a social entity. This paper considers that the values acquire special relevance in a religious institution of public service and delivery to the most disadvantaged and that the interaction between professional and personal roles can be conditioned by the personal profile of human values.

An unquestionable fact is that values play a leading role in organizations with a strong social mission. Hai and Daft (2016) note that in those organizations facing the challenge of combining financial sustainability with social benefits, conflicts are common often, and values underline the solutions. In these organizations, which Hai and Daft (2016) term as hybrids, the environment is characterized by a set of assumptions, beliefs and values that constitute a “logic” that members use to give meaning to their daily lives. In this logic, religious workers should have an intense social perspective, in which collectivism (self-transcendence) is imposed on individualism (self-enhancement). Indeed, Kim’s (2012) study identifies social and religious workers (in addition to managers, natural/social science researchers and medical and allied professionals) as socially minded occupations in Korea.

In this research, a double moderating effect of the values in the work engagement and flourishing relation is expected. On the one hand, we must take into account that nuns do good through working with the poor (whereas other religious orders reach God by other means, for example, through a monastic and contemplative approach). This circumstance should cause the work engagement and flourishing relationship to become more intense as self-transcendence prevails over the desire for self-enhancement (hypothesis 2a). On the other hand, this group’s value set contains features such as obedience, humility, tradition, and social order. This group also has a high average age (common to most religious orders in Europe) and the need for order and stability to keep providing service to the community. All this leads us to think that the desire for conservation will intensify the positive effect of work engagement on flourishing at work (hypothesis 2b).

We thus propose the following research hypotheses:

**Hypothesis 2:** The work engagement and flourishing relationship is moderated by human values such that the autotranscendence-self-enhancement (H2a) indicator and the conservation-openness to change (H2b) indicator reinforce the positive effect of work engagement on flourishing.

**FIGURE 1:**
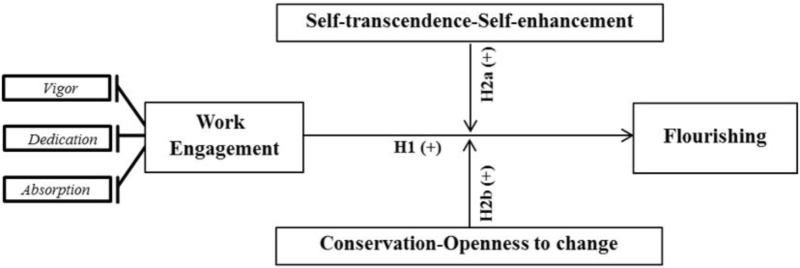
Research model and working hypotheses.

**Figure 1** summarizes the theoretical model and the research hypotheses.

## Methodology

### Sample and Data Collection

According to the objectives of this research, a questionnaire was designed to measure the different variables incorporated into the proposed model. The questionnaire was administered through the Google Form platform, which included a letter explaining the purpose of the investigation. The subjects directly answered to the survey on the platform. The data collection took place between April and May 2016. During that period, the number of consecrated members of the order was 208, and the questionnaire was sent to all of them; 196 valid questionnaires were received, representing a response rate of 94.2%. For sample homogeneity, only the 142 valid questionnaires from to nuns working in the social sector (basically, social canteens, nursing homes and homes for children) were selected for analysis; thus, nuns working in the education sector were excluded.

Although there are many types of religious institutions that profess their faith according to a specific charism, the one under study follows a model of active, non-contemplative life. Its mission is to serve the poor through action, and the charism is developed through work with poor people. The fields of action are education and care for the elderly, children and socially excluded groups. This circumstance serves our purpose since other kind of more contemplative orders would not allow us to deepen in the type of analysis intended in this study.

The majority of the respondents were women (95.0% of the total) (although this research deals mainly with nuns, in the sample there were also a few priests linked to the religious order), with a mean age of 55.2 years (*SD* = 15.8) and an average tenure of 24.8 years (*SD* = 19.2). Regarding educational level, 43.7% of the respondents stated that they had completed university studies, 36.6% had finished secondary studies, and the remaining 19.7% had only studied at the primary education level. Only 7.0% of the respondents lived alone, whereas the remaining 93.0% lived in a community.

### Measurements

The literature review provides the basis for the survey design. The different variables and constructs are measured as follows.

Work engagement is evaluated with the Utrecht Work Engagement Scale (UWES), developed by Schaufeli and Bakker (2003, Unpublished). We have employed the Spanish version of this scale by Benevides-Pereira et al. (2009). Three items measure each of the dimensions of the questionnaire: dedication (e.g., *I am enthusiastic about my work*), vigor (e.g., *At my work, I feel strong and vigorous*) and absorption (e.g., *I am immersed in my work*). The items are measured on a scale ranging from 1 (*never*) to 5 (*always*), so a high score in each of the dimensions indicates a higher degree of work engagement. Different studies, such as Schaufeli et al. (2006) and, more recently, Demerouti et al. (2015), have demonstrated the validity and reliability of this instrument. The reliability estimated in this study for the three dimensions ranged from 0.722 (absorption) to 0.82 (dedication) (see **Table 1**).

**Table 1:** Descriptive statistics, Cronbach’s alpha and inter-correlations for the study variables.

Variable	Mean	*SD*	Cronbach’s alpha	1	2	3	4	5	6	7	8
(1) Vigor	4.33	0.75	0.789	1							
(2) Dedication	4.50	0.72	0.823	0.721^**^	1						
(3) Absorption	4.23	0.80	0.722	0.532^**^	0.639^**^	1					
(4) Flourishing	4.50	0.65	0.879	0.590^**^	0.685^**^	0.537^**^	1				
(5) Self-transcendence	3.77	0.42	0.702	0.337^**^	0.303^**^	0.143	0.246^**^	1			
(6) Self-enhancement	1.97	0.71	0.704	-0.081	-0.033	0.083	-0.143	-0.058	1		
(7) Conservation	3.46	0.55	0.713	0.146	0.105	0.159	0.071	0.361^**^	0.228^**^	1	
(8) Openness to change	2.72	0.65	0.711	0.066	0.150	0.065	0.190^*^	0.130	0.550^**^	0.083	1


*^∗^*p* < 0.05, ^∗∗^*p* < 0.01, ^∗∗∗^*p* < 0.001.*

A subscale of the well-being of Diener et al. (2010) it is used for measuring flourishing. Mendonça et al. (2014) adapted this measure to the work environment. Flourishing is operationalized through eight items that measure the respondents’ self-perception regarding important aspects of human functioning, such as relationships, life purpose life or optimism. All items are expressed in the positive sense. Example items on the scale are the following: *At work people respect me; my work contributes to a meaningful and purposeful life;* and *at work, I contribute actively to the happiness and well-being of others*. Individuals express their degree of agreement with each of these items on a scale of 1 (*strongly disagree*) to 5 (*strongly agree*). The work of Mendonça et al. (2014) demonstrates the psychometric properties of the scale. The Cronbach’s alpha for this scale was 0.879.

To measure human values according to Schwartz’s (1992) Theory, the reduced version (21 items) of the original Portrait Value Questionnaire (PVQ) is used. This instrument is included in the European Social Survey and measures 10 fundamental values (e.g., power, benevolence, tradition…) that are grouped into four higher-order constructs and two orthogonal axes (self-transcendence versus self-enhancement, on the one hand, and conservation versus openness to change, on the other). Each of the items on the questionnaire describes an individual with whom the respondent can identify or not (“*in no way fits the description of me*” = 1, “*the description closely resembles me*” = 4). Some examples of items on the questionnaire are: (a) *It is important to her/him to be rich. She/he wants to have a lot of money and expensive things (power)*; (b) *it is very important to her/him to help the people around her/him. She/he wants to care for their well-being (benevolence*); (c) *it is important to her/him to be humble and modest. She/he tries not to draw attention to herself/himself (tradition)*; and (d) *she/he looks for adventures and likes to take risks. She/he wants to have an exciting life (stimulation)*. Schwartz and Rubel-Lifschitz (2009) demonstrate the validity and reliability of PVQ in different contexts, obtaining reliability indexes ranging from 0.37 to 0.70. Our research obtained an alpha coefficient of 0.702 (self-transcendence), 0.704 (self-enhancement), 0.713 (conservation) and 0.711 (openness to change).

### Methodology: Linear Regression Model

According to the empirical design objectives of this article, several linear regression models are estimated using the forward step method. The complete flourishing model is as follows:

$$\begin{array}{l} Flourishing=\beta_{0}+\beta_{1}Gender+\beta_{2}Age+\beta_{3}\\ Training+\beta_{4}Tennure+\beta_{5}Vigor+\beta_{6}Dedication\\ +\beta_{7}Absorption+\sum_{i=1}^{10}\beta_{8i}(Vigor\;\times \;Values_{i})\\ +\sum_{i=1}^{10}\beta_{9i}(Dedication\;\times \;Values_{i})+\sum_{i=1}^{10}\beta_{10i}\\ (Absorption\;\times \;Values_{i})+\varepsilon \end{array}$$

“*i*” are Values of Schwartz theory: Power, Achievement, Hedonism, Stimulation, Self-Direction, Universalism, Benevolence, Conformity, Tradition and Security.

NOTE: **Table 2** contains the meaning of the variables.

**Table 2:** Flourishing regressions.

	Model 1	Model 2	Model 3
Constant	3.864	1.665	2.205
Gender	0.179	-0.014	-0.119
Age	0.006	0.039	0.101
Education			
Middle	0.204	0.043	0.075
Upper	0.249	-0.099	-0.199^*^
Tenure	0.000	0.022	0.153
Vigor		0.187	0.115
Dedication		0.625^***^	0.476^***^
Absorption		0.173	0.136
Vigor × (Self-Transcendence–Self-Enhancement)			-0.165
Dedication × (Self-Transcendence–Self-Enhancement)			-0.370
Absorption × (Self- Transcendence–Self-Enhancement)			0.040^**^
Vigor × (Conservation-Openness to Change)			-0.176
Dedication × (Conservation-Openness to Change)			-0.034^*^
Absorption × (Conservation-Openness to Change)			0.767
**Adjusted *R*^2^**	**0.042**	**0.464**	**0.418**


*^∗^p < 0.05, ^∗∗^p < 0.01, ^∗∗∗^p < 0.001. All numbers reported are the standard regression coefficients. Reference categories: male, low education.*

## Results

### Descriptive Statistics

With regard to the central variables in this study, **Table 1** presents the main descriptive statistics, as well as the Cronbach alpha for each analyzed construct, and the bivariate correlations between the main research variables. As it can be observed, the consecrated members of the order seemed to be quite “engaged” in their work, with values close to the maximum level of 5 in each of the three dimensions of the construct (vigor: 4.33; dedication: 4.50, absorption: 4.23). In addition, they show a high level of flourishing, i.e., 4.50 of a maximum of 5 points.

The nuns’ human values profile stands out in initial indexes for their high self-transcendence; their two highest scores are in universalism (3.79 out of 5) and benevolence (3.74). They also score high in the conservation dimension: tradition (3.65), security (3.40), and conformity (3.30). At the opposite end of the scale of values, they give less importance to values related to openness to change (stimulation, 2.56, hedonism, 2.56, and self-direction, 3.06). Self-enhancement is the lowest score, at 1.83, followed by achievement, at 2.12. These results are consistent with the values that support the institutional discourse. In summary, nuns prioritize self-transcendence (3.77) more than self-enhancement (1.97), and they are more conservative (3.46) than predisposed to change (2.72).

**Table 1** also reveals that the main variables are significantly related between each other, which is consistent with the most relevant research theories and hypotheses mentioned above.

### Regression Models

The results are presented in several stages including different linear regression models. The first one introduces some sociodemographic variables that the previous literature has related directly to flourishing (see, for example, the works of Keyes, 2002; Gokcen, 2013). In the second stage, the three variables that make up the construct work engagement (vigor, dedication and absorption) are introduced into the model to analyze how this phenomenon affects flourishing. Finally, Model 3 includes the moderating effect of values on the work engagement and flourishing relationship through two indices that synthesize the four main dimensions of Schwartz’s (1992) Theory. No multicollinearity has been observed in any of the models.

The results are shown in **Table 2**. As observed in Model 1, none of the sociodemographic variables contributes to explaining the level of flourishing of nuns.

According to model 2, flourishing is basically explained by the effect of dedication to work. This model accounts 46.4% of the variance in flourishing. The entry of one of the three dimensions of work engagement to the model empirically confirms the interrelation between personal and professional roles among the consecrated members of religious orders. This result corroborates the first hypothesis of this research.

The second hypothesis proposes the existence of a moderating effect of human values that permeate the religious spirit in the work engagement and flourishing relationship. To address this question, two indicators are estimated that represent the relative position of the respondents in the two large bipolar dimensions that Schwartz’s Theory contains. On the one hand, the pole facing continuity versus the desire for change is calculated by the difference between the individual scores of the Conservation and Openness to change (C-OC) higher-order constructs. On the other hand, the pole that contrasts collectivism with individualism is obtained by the difference between the higher-order constructs of Self-Transcendence and Self-enhancement (ST-SP). The moderating effect of human values on the work engagement and flourishing relationship is determined as the product of these two indices for each of the dimensions of work engagement.

Upon the introduction of the moderating effect of values, the multivariate model yields significant results on the two axes that form the basic structure of the theory proposed by Schwartz, adding to previous ones. The moderating influence of human values on the work engagement and flourishing relationship is shown, on the one hand, through the absorption dimension combined with the self-transcendence/self-enhancement axis and, on the other hand, through the dedication dimension combined with the conservation versus open to change axis. In this stage, dedication to work remains exerting a strong effect in the work engagement and flourishing relationship. In addition, higher educational level emerges in the multivariate model because of its direct relationship with flourishing.

These results confirm our second hypothesis. The variables that make up Model 3 jointly explain 41.8% of the variance in flourishing.

## Discussion

First of all, it should be noted that our findings are useful for organizations in which individuals or leaders feel their work as a call (Dempsey and Sanders, 2010) and in which flourishing is achieved through personal involvement in the project. In other contexts, the positive effects of existential well-being on job satisfaction have been demonstrated (Robert et al., 2006), so there is a feedback for vocational workers. In this sense, different researchers, such as Cotton et al. (2003) and Parker et al. (2008), consider religious workers a distinct occupational cohort among helping professions, a group that experiences a unique combination of motivations, changes, resources and demands. For example, the study of Sav et al. (2011), carried out with a sample of Australian Muslims, suggests that human values that characterize this group indicate that the interrelation between the labor role and the personal role is different from that of the general population in Australia. The origin of this work intended to answer two basic research questions related to this special group of employees. One, is there a direct relationship between work engagement and the level of flourishing of members of a religious organization in Europe? Two, is there a moderating effect of the human values of nuns in the work engagement and flourishing relationship?

### Direct Relationship Between Work Engagement and Flourishing

Rosenbaum et al. (2011) emphasize the role of well-being in career development, and more specifically, some papers have related work engagement with well-being (Diener et al., 2003; Salanova et al., 2003; or more recently Halbesleben, 2010; Bakker and Oerlemans, 2011). However, we have not detected any research that has paid attention to a context as unique as that of religious organizations, and no study has included the moderating effect of the human values that characterize the members of a Catholic congregation. The work that religious workers do to serve the poorest people can be highly rewarding. Simultaneously, their daily activity is carried out in a stressful, frustrating and discouraging environment, with old people who die, children who are forced to return to unstructured homes, and drug addicts who fall again into the abyss of the drug. Additionally, they must perform tasks outside their religious vocation (administrative, bureaucratic, volunteer management, etc.) for which, according to Bickerton et al. (2014), they do not consider themselves prepared, and they feel that these activities distract them from their true spiritual mission, i.e., to serve the poorest people.

Considering the purpose of our research, it is relevant and timely to use human flourishing as a measure of well-being. In this sense, Seligman (2002) notes that the core of personal well-being is made up of elements including engagement and interest. As indicated by Steger et al. (2008), the theoretical and empirical studies on this theme highlight the importance of meaning and purpose in life in the pursuit of well-being. Finally, we also believe that introducing the moderating effect of human values according to Schwartz’s (1992) Theory helps fill an important research gap, thereby contributing to improving scientific knowledge on the topic.

In a first stage, this work tried to identify whether nuns’ degree of engagement with their activities is related to their level of flourishing. Adopting the analysis strategy of Oishi et al. (1999), who postulate that people ponder value-congruent domain satisfactions more heavily than value-incongruent domain satisfactions, the second stage of this investigation explored whether the work engagement and flourishing relationship was inhibited or reinforced by the profile of personal values that permeate the spirit of nuns.

**Table 2** shows the most relevant results. Model 1 displays that none of the estimated coefficients of the flourishing equation is statistically significant, which means that factors of a personal nature among the religious group do not explain their flourishing. These results are inconsistent with other studies that have found a greater degree of flourishing among married (an affair that is not possible among nuns for obvious reasons) and educated adult males (e.g., Keyes, 2002; Clark and Senik, 2011).

The low explanatory power of the model changes substantially by incorporating in Model 2 the different variables that are relevant to the aims of this research, i.e., vigor, dedication and absorption, the three dimensions that define work engagement. In this case, the explanatory capacity of the model reaches 46.4%, and all of the variance is explained by a single independent variable: dedication to work. This result is consistent with some previous studies that find a strong positive correlation between job dedication and satisfaction (Vansteenkiste et al., 2007). As Bakker (2011) suggests, highly engaged workers are more productive and are always willing to give a little more. Following Wrzesniewski (2003), considering job as “a calling” will allow people to work more. In the specific field of well-being, some studies have found a direct relationship between dedication at work and the hedonic component of well-being, i.e., satisfaction with life (see Schaufeli et al., 2008; Hakanen and Schaufeli, 2012; for example, Rothmann, 2008). However, a recent study finds no such relationship between dedication to work and satisfaction with life in a sample of South Korean hotel employees (Lee et al., 2016).

As indicated above, there is little scientific literature to support the results of the present study in the specific context of faith-based organizations that provide social service to the community. This is despite the fact that researchers such as Ellison (1991) have noted the influence of religious variables on well-being, in the sense that the deterioration of well-being because of the presence of labor stressors should be offset by the positive effect of religiosity on employees’ minds. Certainly, religious beliefs bring coherence and order to life, which somehow translate into greater well-being, both physical and psychological. Although they operate in a highly heterogeneous work context, a recent study by Demerouti et al. (2015) shows that the motivation of religious people leads them to flourish. These authors emphasize that work engagement plays a protagonist role when developing that internal motivation component where the behavior is performed for itself, for the mere fact of experiencing the pleasure and the enthusiasm inherent in the work activity. Undoubtedly, the most engaged religious workers will be particularly motivated to activate or create labor resources (e.g., through training, soliciting support from colleagues or feedback from superiors) to enable them to cope with the multiple tasks that they must attend in both the spiritual and material spheres (Bickerton et al., 2014).

In summary, the conclusions drawn from the results of Model 2 confirm the approach of our first research hypothesis, suggesting that nuns can feel good through dedication to the work. This reasoning, which can be extrapolated to most occupations and justifies the interactive effect between the domains of work and personal life, is especially relevant among religious workers, since the line separating work from personal life is virtually non-existent. As proposed by Johnson (2010), while religion erases the boundaries between public and private life, it simultaneously provides a mechanism of integration that contributes to reducing the tensions of contemporary life.

However, although each nun could have her own interests, the results display a low standard deviation in both independent variables; therefore, what unites them is greater than what separates them and. From this point of view, the strength of the work-well-being relationship will be shaped by the way in which such a link fits, or not, with her vital expectations and with her professional activity. For religious workers, their work is part of their vital expectations, as many religious orders display their vital mission in actions with others. By living faith in everyday life, the border between the professional sphere and the vital sphere is blurred.

### Moderating Effect of the Human Values in the Work Engagement and Flourishing Relationship

Given the direct influence of work on flourishing, the second research hypothesis stated that there are plenty of reasons to think that this relationship is conditioned by nuns’ human profile, their values and their intimate feelings toward their profession. Undoubtedly, working conditions and the demands of the workplace can exert a direct influence on their flourishing, and this influence will be conditioned by the degree or content of certain personal values. Working in such an absorbing labor context, as described in this paper, requires an important degree of generosity and altruism. Therefore, it is logical to think that the work engagement and flourishing relationship is intensified among those nuns who prioritize collective welfare (social justice, equity, peace, honesty, i.e., self-transcendence) over their own personal interests (self-enhancement). This circumstance suggests that values can play a moderating role in the work engagement and flourishing relationship. In this scenario of coherence, something similar should manifest among nuns with more conservative human values.

In Model 3, we introduced an interaction term between the different dimensions of this construct (vigor, dedication and absorption) and the corresponding indices to the two orthogonal axes of Schwartz’s Theory (self-transcendence and self-enhancement, on the one hand, and conservation versus openness to change, on the other). The results of Model 3 confirm that human values moderate the direct influence of work engagement on flourishing. There is a positive and significant coefficient of the interaction between labor absorption and the self-transcendence index. It indicates that the link between labor absorption and flourishing is more intense for more altruistic nuns, i.e., those who develop a human behavior of greater disinterested attention toward others, those who care about social justice, those who are more tolerant or those who aim to address inequalities, among other defining values of their personality. This fact is even more pronounced in comparison with other nuns who are also interested in power and personal achievement. This result is consistent with the approach of Cunha et al. (2014), who consider that the presence of Christ in organizations has an impact on managerial attitudes by creating a more humane working community and an orientation toward virtue. The statistically significant expansive effect of altruism on the absorption-flourishing relationship may be motivated by the fact that nuns’ primary source of personal well-being comes from absolute an immersion in certain professional activities (as teachers, nurses, geroculturists, cooks) that require attitudes toward public service and a level of abnegation and innate generosity toward others. All this implies, as Van den Bosch and Taris (2014) note, full concentration and absorption in work, to the point that the day passes quickly, almost without employees realizing it, and they find it difficult to leave their post and go home. The importance of self-transcendence in the religious context is evidenced in the work of Oberholster et al. (2013), who conclude that it is the main motivational factor for expatriate workers in faith-based humanitarian organizations, and in the qualitative research of Monteiro (2015). In a sample of Catholic leaders, this author notes that acting guided by the love of God results in a greater involvement with different stakeholders. That is to say, an organization benefits as altruism leads to mutual profit and understanding instead of competition.

Regarding the conservation-openness to change axis, the negative and significant coefficient obtained in the multivariate equation of Model 3 suggests that the link between dedication to work and flourishing is less intense among the more conservative nuns (those who are more moderate and disciplined, who aspire to order and maintain the status quo) than those most predisposed to change. This result contradicts what was predicted in the second research hypothesis, where a multiplier effect of work engagement on flourishing was expected among nuns who presented a more conservative human values profile. In this work, two possible explanations are explored. The first is that the possible autonomy of highly motivated workers, such as expatriates from humanitarian organizations, has a positive effect on their satisfaction with life, if they rely on the support of managers (Visser et al., 2016). Therefore, the relationship of brotherhood among the nuns is the appropriate framework for trusting autonomy as an organizational tool to improve life satisfaction. Second, dedication constitutes a dimension of work engagement related to a sense of meaning, enthusiasm, inspiration, pride and challenge (Schaufeli and Bakker, 2003, Unpublished). Consequently, the stability associated with conservative values prevents adequate personal growth through work. Additionally, it must be added that social denunciation is located in the deeper molecules of the DNA of the investigated congregation. Social denunciation is the desire to modify those unfair structures and systems that generate and perpetuate the main social problems that are opposed by nuns, e.g., hunger, marginalization, inequality, and poverty. This circumstance can modify the negative moderating effect of dedication to work on the flourishing of more conservative nuns.

A couple of observations should be noted in the third model. First, dedication to work continues to have a direct effect on flourishing. Second, a personal variable emerges in the multivariate model because of its direct relationship with flourishing. Religious workers with a higher educational level are precisely those who exhibit a lower level of flourishing. This relationship is of great interest since most studies on this subject have concluded that further training is an essential component of well-being (a critical view on this issue can be found in Michalos, 2008). Expectations of more uncertain fulfillment may generate frustration and disenchantment among some nuns when they perceive that they are not able to fulfill everything that is expected of them.

## Conclusion

Two significant conclusions are derived from this work. First, the more engaged nuns are in their work (social action to serve the poorest and most disadvantaged people), the more they flourish in their working environment and in their personal lives. The desire to dedicate physical, cognitive and emotional resources to work (Christian et al., 2011) is particularly deep among nuns, as they also give themselves spiritually to the cause to which they have decided to offer their lives. Second, certain personal values reinforce the relationship between the professional role (work engagement) and the personal role (flourishing).

In conclusion, it should be noted that this study has been able to analyze the members of a Catholic religious order, something that is not easy in a world as secularized as the present one – particularly in the European context, an environment where religion (and religious people) are sometimes observed with too much suspicion. This hostility is hardly understandable due to the valuable contribution of these institutions to certain sectors of activity critical to the maintenance of the welfare state, such as health, education or social action (disability, immigration, drug addiction, and child abuse). Using a religious sample, this study offers a series of relevant results that contribute significantly to the debate about work engagement and flourishing interaction and clarify the impact that human values exert on this phenomenon. The relationship between personal and professional life is an issue that has been given considerable attention from studies with different approaches (gender approach, welfare policy or harmonization of working hours, among others). However, this topic has undoubtedly been hardly addressed in this collective, as if it were assumed that nuns, simply because they are nuns, have no right to a private and personal life.

## Implications and Limitations

This research has significant practical implications from inquiring into the deepest values and feelings of human beings. First, this study offers researchers empirical evidence about how work engagement improves individual well-being. It also shows that human values moderate the relationship between work engagement and flourishing in a group of people, nuns, who exercise moral leadership in their organizations. Second, the present study contributes to the general body of research in vocational employees and particularly on the religious workers’ role and the nature of their activity. The results of this study provide the Order’s Curia with a better awareness and understanding of what religious workers need in order to provide an adequate environment as well as other benefits and/or training programs that contribute to their satisfaction and commitment. In particular, attention should be paid to the overload of work to which nuns are subjected on many occasions, especially in administrative-bureaucratic activities that distract them from their authentic mission, i.e., to serve the poorest and most disadvantaged people. Only in this way will nuns achieve a significant improvement in flourishing achievement. Third, as with many other religious orders in Europe, the high average age seriously compromises the future of the institution given the lack of religious vocation in community. In the near future, non-religious collaborators will engage deeply in the management of the 80 establishments that the order controls in the South of Spain. The management of these new successors in the mission may improve understanding how the values moderate the relationship between work engagement and flourishing.

Finally, other settings in which leaders present vocational characteristics such as the one we have studied could be that of entrepreneurs.

Finally, some precautions must be adopted interpreting the results. The main methodological limitations of this study are as follows: (1) a causal relationship cannot be established between the variables of the present research given the transversal nature of the data; (2) there can be a response bias motivated by the problem of social desirability, given the sensitivity of the subject investigated, human values, and the method of obtaining information, i.e., self-reports; (3) the field of study was a single religious order located in a very specific geographical area, which might represent a drawback. Further research should cover other religious institutions, analyze the influence of different cultural contexts, and extend the scope of study to lay employees who share with religious workers the difficulties of day-to-day work. A final limitation of this study is that it has not been possible to analyze the age effect on work engagement due to the high degree of concentration of the sample in the highest age strata. For example, 73% of the sample was older than 45 years, and 22.2% were 70 or older, a circumstance that corresponds to the reality of most Catholic religious orders.This is a limitation since literature on social identity shows that employees who are engaged with their organizations are more prone to commit themselves in voluntary behaviors. The reason is that they feel their organizations are an important part of their self-definition [on this topic you can see, for example, the work of Avanzi et al. (2012), conducted with a sample of Italian teachers]. Although it is not easy given the scarcity of vocations in the Catholic Church, our intention in future research is to increase the size of the sample with younger religious to confirm if age increases or not the likelihood that nuns develop a strong sense of belonging as happens in other professions with a less vocational substratum than that of the religious orders.

## Ethics Statement

This study was carried out in accordance with the Declaration of Helsinki, and the protocol was approved by the Ethics Committee of Universidad Loyola Andalucía (Spain). All subjects gave written informed consent.

## Author Contributions

All authors listed have made a substantial, direct and intellectual contribution to the work, and approved it for publication.

## Conflict of Interest Statement

The authors declare that the research was conducted in the absence of any commercial or financial relationships that could be construed as a potential conflict of interest.
